# "Do we register our deaths?": Motivations and barriers to death registration in Ghana

**DOI:** 10.1371/journal.pone.0312353

**Published:** 2024-10-24

**Authors:** Martin Wiredu Agyekum, Frank Kyei-Arthur, Seth Kissi Bosompem

**Affiliations:** 1 Institute for Educational Research and Innovation Studies, University of Education, Winneba, Ghana; 2 Department of Environment and Public Health, University of Environment and Sustainable Development, Somanya, Ghana; 3 Regional Institute for Population Studies, University of Ghana, Legon, Ghana; 4 Births and Deaths Registry, Accra, Ghana; The Hong Kong Polytechnic University, HONG KONG

## Abstract

Death registration is generally low in sub-Saharan African countries, including Ghana. This study investigated the factors that motivate and serve as barriers in registering for a death certificate. A cross-sectional qualitative study design was used to interview twelve birth and death registration officers in Ghana. The interviews were analysed using Atlas.ti version 7.5. The results show that death registration in Ghana is generally low. Processing of a deceased person’s estate emerged as the main motivation for the registration of death. Other motivators included the obligation to register deaths and the requirement of death certificates for burial. The barriers to death registration included the governance system at the cemetery, the hastiness in burying the dead, the perception of cost of death certificates, and less importance attached to death registration. Policymakers should consider these factors in the design of interventions to increase the rate of death registration in Ghana.

## Introduction

The United Nations defines civil registration as the "continuous, permanent compulsory and universal recording of the occurrence and characteristics of vital events on the population as provided through decree or regulation following the legal requirements of a country" [1]. These include recording vital events such as births, deaths, marriages, and divorces [2]. An effective civil registration system establishes the legal identity of individuals and provides regular information on demographic trends, including mortality and cause of death [3]. Most developed countries have relatively better organised systems to record deaths [3]. According to the World Health Organization [4], death registration is generally low in sub-Saharan African countries for the period 2007–2016, except for Mauritius (98%), Cabo Verde (92%), South Africa (92%), and Seychelles (91%), which have high completion rates of death registration due to several factors, including comprehensive system of death registration, technological advancement in health information systems, better health infrastructure, and strong government commitment. In terms of data quality of cause-of-death, Mauritius’s data were of high quality, while that of Cabo Verde were low.

Death registration or certification provides valuable health information on the cause of death, such as disease incidence and prevalence [5,6]. A reliable and timely pattern of mortality patterns guides local health policies and decision-making to ensure that resources are prioritised and managed effectively and efficiently [7,8]. On the one hand, death registration is beneficial for individuals and countries [9]. For instance, a death certificate is required to access a deceased person’s social and economic benefits [9]. It also helps families provide data on causes of death, which may help identify hereditary diseases and help family members implement measures to reduce their risk of these diseases [7]. Countries are also required to annually document births and deaths in their populations to ensure the effective operation of their health systems [10]. Ineffective or non-existent death registration systems can have social and economic implications [9]. For example, governments may be constrained in their ability to sanitise payrolls, breeding an opportunity for fraud and a high public sector wage bill. Additionally, poor death registration systems can affect the quality of population-level data used for planning and resource allocation, all of which have potential negative financial and economic impacts [9].

Several factors have been identified that contribute to the motivations for and barriers to death registration. Evidence shows that both micro- and macro-level factors, such as public awareness and structural factors (e.g., unavailability of registration officers, lack of political will, limited use of technology, and poor attitudes of registration officials) influence death registration [11,12]. In addition, socio-demographic and economic factors have been identified to influence death registration [8,9].

In Ghana, the Births and Deaths Registry is responsible for registering deaths. The Births and Deaths Registry is established by the Births, Deaths, and Burial Ordinance of 1912 [13]. However, death registration began much earlier, having been established by the Cemeteries Ordinance of 1888 [14]. Death registration depends on whether (a) the death happened at a health facility after 24 hours of admission or (b) outside the health facility or before 24 hours. To record a death that occurred in a health facility after 24 hours, a relative must visit the district office of the Births and Deaths Registry in the area where the death took place. Additionally, the relative must give the Registrar the original medical cause of death certificate and provide the deceased’s name, age, address, and informant’s name and relationship with the deceased [2,15].

However, if the death occurs outside a hospital or before 24 hours, a relative must notify the nearest police station about the death. Police will investigate and file an inquest with the nearest court. Once the court issues a coroner’s report, the deceased’s relative will submit an original copy to the district office of Births and Deaths Registry where the death occurred. The deceased’s relative will also supply the necessary information to register the deceased in the death register, including the deceased’s name, age, address, informant’s name, and relationship with the deceased [2,15]. Several efforts, such as public awareness and sensitisation programmes, have improved birth and death registration [16]. Despite all these efforts, death registration is very low compared to birth registration in Ghana [17,18]. It is estimated that 60–65% of deaths in Ghana are unrecorded each year [17].

There are few studies on factors associated with death registration in sub-Saharan Africa (SSA) [3,9,19,20]. However, to the best of our knowledge, no study examining the barriers and motivations for death registration in Ghana has been conducted. The limited studies on civil registration in Ghana have focused on birth registration [16,21,22]. In addition, the few studies on death registration in SSA used only quantitative methods; therefore, they do not provide an in-depth understanding of the factors that act as motivators for and barriers to death registration. This study, therefore, examined the facilitators and barriers to death registration in Ghana using a qualitative method. The findings of this study will inform policymakers on strategies to improve the registration of deaths in Ghana and SSA.

### Theoretical framework

This study hinges on the theory of planned behaviour to understand the motivators for and barriers to death registration in Ghana. The theory postulates that individuals’ actions or behaviours are influenced by their attitudes, subjective norms (perceived social pressure), and perceived behavioural control [23]. Thus, these factors regulate people’s actions or behaviours, which may influence their decision to either register or not register the dead for a certificate.

## Materials and methods

### Study design and sampling procedure

A qualitative study design was used to explore motivators for and barriers to death registration in Ghana. This study is part of a larger study that assessed factors that influence birth and death registration in Ghana.

This study applied a two-stage sampling process to select the interviewees. The first stage involved the selection of study areas. For the first stage, we purposively selected three regions and six districts, as study sites. The country was divided into three ecological zones. These zones are the Coastal, Middle, and Northern zones. One region was selected from each ecological zone. Greater Accra was chosen from the Coastal zone, the Bono region in the Middle zone, and the Savanna Region in the Northern zone.

Furthermore, the Districts/Municipalities/Metropolitan regions were selected based on the rural and urban differences. For Greater Accra, Shai Osudoku District, and Accra Metropolitan Assembly were chosen for this study. Sunyani Municipality and Berekum West District were selected in the Bono region. Sawla-Tuna Kalba and Central Gonja were chosen from the Savanna region.

The second stage involved the selection of interviewees in the study. Purposive sampling was used to select interviewees from the six selected districts. Two birth and death registration officers were chosen for the interviews in each district. Hence, four (4) registration officers were interviewed in each region. They were selected based on the following criteria: they must be a full-time employee, having worked for at least 5 years with the Births and Deaths Registry. In all, twelve (12) registration officers were interviewed for this study.

### Study setting

Ghana has 16 administrative regions, and the study was conducted in the Greater Accra, Bono, and Savanna regions. The Greater Accra region, located in the South of Ghana, houses the national capital city, Accra, and is the most populous region according to the 2021 Population and Housing Census, with a population of 5,455,692 [24]. The Accra Metropolitan Assembly and Shai Osudoku District were selected for the study within the region. The Bono region is located in the middle belt of Ghana and has Sunyani as its regional capital town. The Bono region has a population of 1,208,649 as of 2021 [24], and within the region, Sunyani Municipality and Berekum District were selected for the study. In addition, the Savanna region, located in the Northern part of the country, was selected. The region is purely rural compared to Bono and Greater Accra, which are predominantly urbanised. The Savanna region has a population of 653,266 as of 2021 [24].

### Data collection

The data collection was conducted in May 2023 using a semi-structured interview guide developed and piloted for the study. The interview guide contained information on the trends, motivators, and barriers to death registration. The interview guide was reviewed during the training of interviewers and after the pre-testing. Two interviewers with at least 5 years of experience in qualitative research methods were recruited to assist with data collection. A day of training was held to train interviewers on data collection, and it covered topics such as the nature and purpose of the study, ethical data collection, and how to do community and household entry.

Birth and death registration officers were selected for the interviews through the following process. Birth and death registration officers in the six selected districts were contacted, and the purpose of the study, the general objectives, benefits, and risks of taking part in the study were explained to them. Birth and death registration officers who were willing to participate in the study were eligible to be interviewed. In districts where more than two officers expressed interest in participating in the study, the first two officers who expressed interest were interviewed since the study was interested in interviewing two birth and death registration officers in each of the six selected districts.

Written consent was obtained from those who agreed to participate in the study. In addition, interviewees were informed about the strict confidentiality and anonymity of the information they would provide. The interviews lasted an average of 30 minutes and were conducted in the interviewees’ preferred language, mainly English. The interviews were tape-recorded. The Ethics Committee for the Humanities granted ethical approval for the study at the University of Ghana with clearance number ECH 114/22-23.

### Data analysis

The interviews were transcribed verbatim in English. Data quality checks were performed by reading the transcripts and playing the audio tapes to verify that all the audio files had been transcribed correctly. The data were analysed using Atlas.ti analytical software version 7.5 and the analyses looked out for emerging themes. All authors were involved in the data analysis. This study used an inductive analysis method; hence, codes were assigned to interviewees’ narratives as captured in the transcripts, which were related to the aims of the study. First, all the authors read through the transcripts thoroughly to identify the initial codes and themes (Braun & Clarke, 2006). The initial codes were combined to generate the various themes. Thus, similar codes were combined to create basic themes. Various basic themes that reflect similar ideas were combined into organising themes, and all the organising themes were combined to generate a global theme. All the authors agreed on the codes and themes to ensure they reflected the interviewees’ accounts.

The trustworthiness of findings is crucial in qualitative studies [25,26]. Various strategies can be employed to enhance the trustworthiness of qualitative studies, including data saturation, peer review, prolonged engagement, thick description, and audit trail [25]. In this study, the first author analysed the data, and the second and last authors reviewed the themes generated from the data. The second and last authors’ feedback were used to improve the themes. Also, all decisions made during the data collection and analysis were documented to enhance the trustworthiness of the study.

Furthermore, previous studies have demonstrated that data saturation can be achieved with twelve (12) interviews [25,27]. Therefore, this study interviewed twelve (12) interviewees, which helped us to get adequate sample size to achieve data saturation. After analysing the eleventh transcript, no new information or theme emerged.

## Results

### Characteristics of interviewee*s*

Table 1 presents the background characteristics of the interviewees. A total of 12 birth and death registration officers were interviewed. Eight (8) out of the 12 birth and death registration officers were males, while 4 were females. Most male interviewees (75.0%) were aged 31–40. However, an equal proportion of female interviewees were aged below 31 (50.0%) and 31–40 (50.0%). Also, most male (87.5%) and female (75.0%) interviewees had tertiary education. Furthermore, all female interviewees were married, while most male interviewees (62.5%) were never married.

**Table 1:** Background characteristics of the respondents.

	Total		Male		Female	
Characteristics	Frequency	Percentage	Frequency	Percentage	Frequency	Percentage
**Age**						
Below 31	2	16.7	-	-	2	50.0
31–40	8	66.6	6	75.0	2	50.0
41–45	2	16.7	2	25.0	-	-
**Educational level**						
Secondary/Middle	2	16.7	1	12.5	1	25.0
Tertiary	10	83.3	7	87.5	3	75.0
**Marital status**						
Currently married	7	58.3	3	37.5	4	100.0
Never married	5	41.7	5	62.5	-	-
**Total**	**12**	**100.0**	**8**	**100.0**	**4**	**100.0**

Source: Fieldwork., 2023.

### Main themes

**Fig 1:**
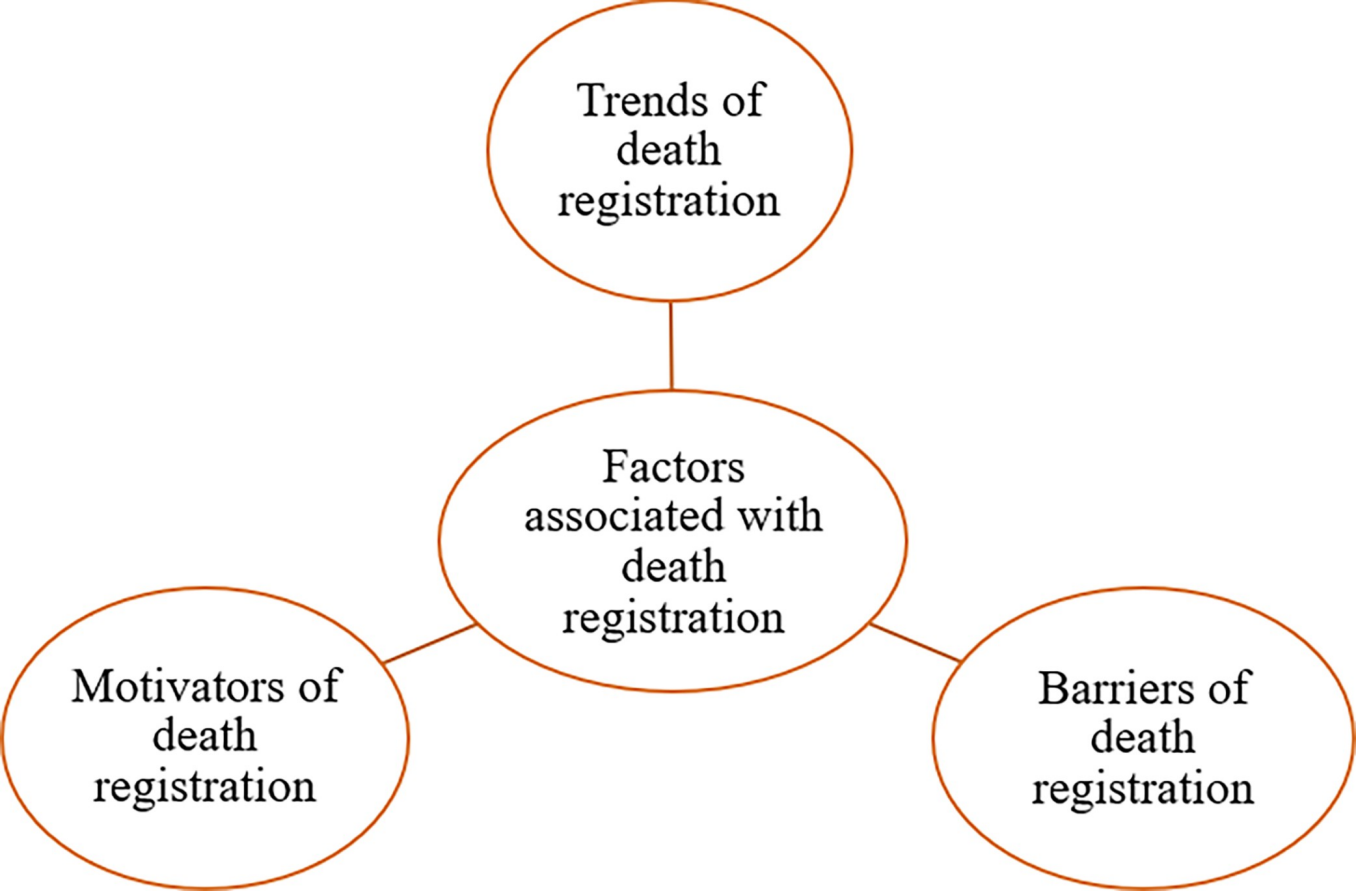
Thematic network showing factors associated with death registration.

Three main thematic areas describe the situation of death registration in Ghana (Fig 1). These were (i) trends in death registration in Ghana, (ii) motivators for death registration, and (iii) barriers to death registration. The sub-themes associated with each organising theme are presented under each theme.

#### Trends in death registration

**Fig 2:**
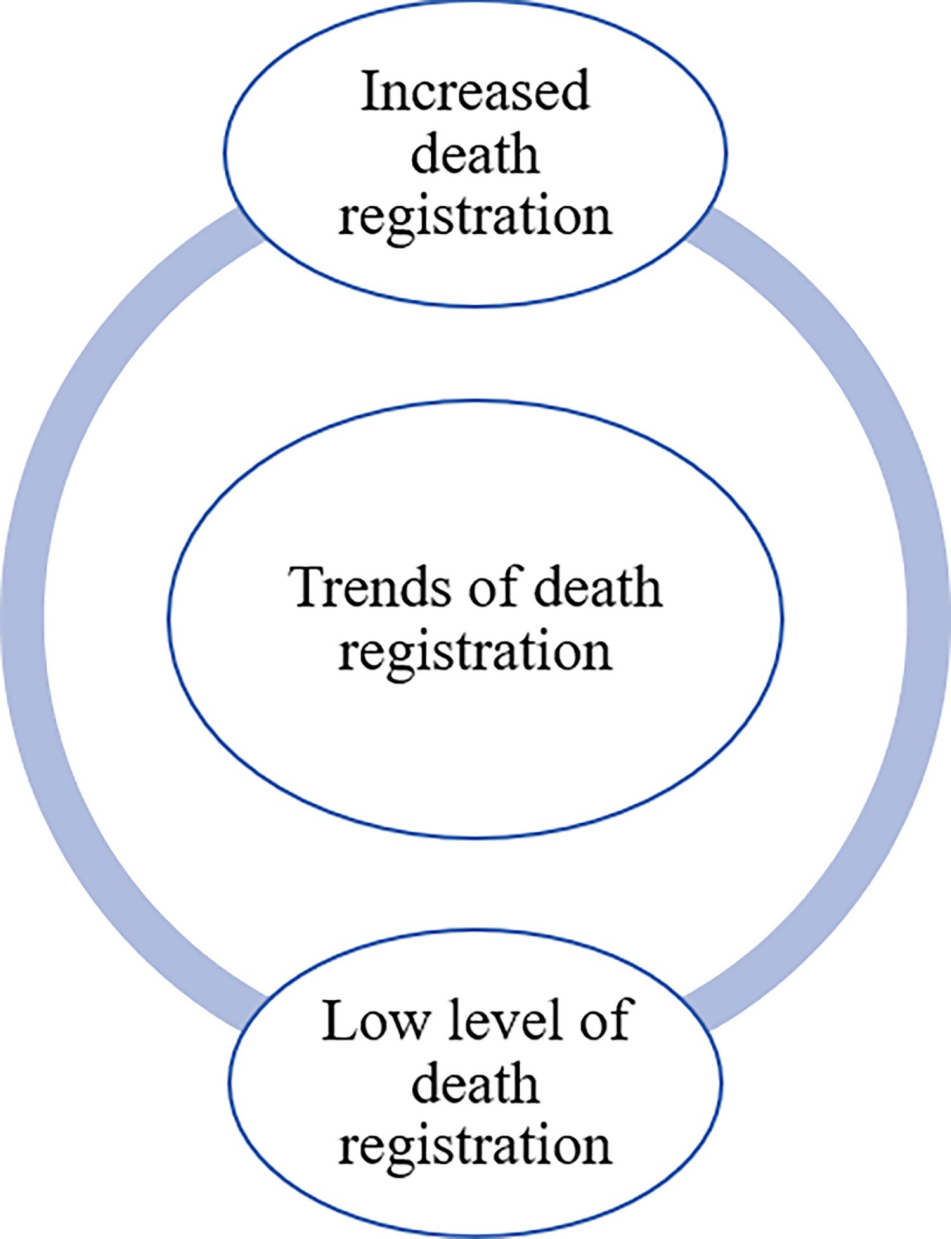
Thematic network showing trends of death registration.

Trends in death registration had two main basic themes: (a) increased death registration and (b) low level of death registration (Fig 2). The basic themes are described below.

*Increased death registration*. Few interviewees expressed that death registration has increased in their districts due to burial requirements at the cemetery. The organised system in the cemetery has facilitated the need for the registration of death and burial permits. Interviewees explained that the increase in death registration was recorded mostly in the regional capitals.

"***I:*** *How is death registration in this district?* ***R:*** *Yes. It is fine.* ***I:*** *Has it increased or decreased?* ***R****: It has increased. In fact, the system or the cemetery is helping. They have an organised system here at the cemetery and the morgues. They encourage people to register their deaths before burial."* (R5, Male)

Another interviewee mentioned that sensitisation programmes by the birth and death registration officers has improved death registration. Through those programmes, people have recognised the importance of death certificates, which thereby has led to an increase in death registration.

"*Death registration is high because in this place [district], the number of people who are buried outside this district is 1 percent. Most of the people who die here are buried either in Korle Gorno or Awudome, so you cannot escape. You will have to register definitely. Additionally, the public education and other activities has increased death registration,."* (R3, Male)

The use of death certificates as a primary document to process other documents for a deceased person has necessitated the need for people to register their deaths.

"*Okay, I would say yes, it is better than previous times. Because right now, we are in a kind of a state where before you process any kind of document for the death of an individual, the primary document you need is the death certificate so that encourages people to chase the death certificate* ***I:*** *okay* ***R:*** *so, it has greatly improved* ***I:*** *so, looking at the trend it has increased or decreased* ***R:*** *it has increased."* (R6, Male)

*Low level of death registration*. According to the narratives, low death registration has been recorded in most of the districts. Interviewees reported that there are few registration centres, which are often diversely distributed. Consequently, the distance to these registration centres is long, which contributes to low death registration. The long distance is compounded by the high cost of transportation from some communities to the registration centre. One interviewee expressed that the transportation cost sometimes discourages people from death registration.

"*Here, death registration is low, I mean, I do not know how to say it, but it is not plenty. In a month, you can get about 5 death registration."* (R7, Female)

*"****I***: *Is death registration high or low in the district*? ***R*:** *I will say it’s low*. ***I***: *Why do you think it’s low*? ***R***: *The factors I gave*, *you know*, *somebody travelling from a village in Agortor to do registration and*, *say*, *our district office registry at Asutsuare*. *The transportation cost for the return trip will cost the person 100 Ghana Cedis (US $ 10)*. *They get discouraged to travel to (laughs) do those things*.*"* (R1, Female)

Although there have been some sensitisation programmes in some of the communities in the Northern and Southern parts of the country, the registration of deaths, especially in the Northern part, continues to be low because most residents do not know the relevance of death certificate registration.

"***R****: Death registration is very poor because they do not value death certificates here. They do not know its importance. Even though sensitisation is ongoing, some residents will tell you the person is dead, so what am I going to register the death for? You see unless you take your time as an officer to explain things to the person, they would not appreciate its importance."* (R9, Male)

#### Motivators

**Fig 3:**
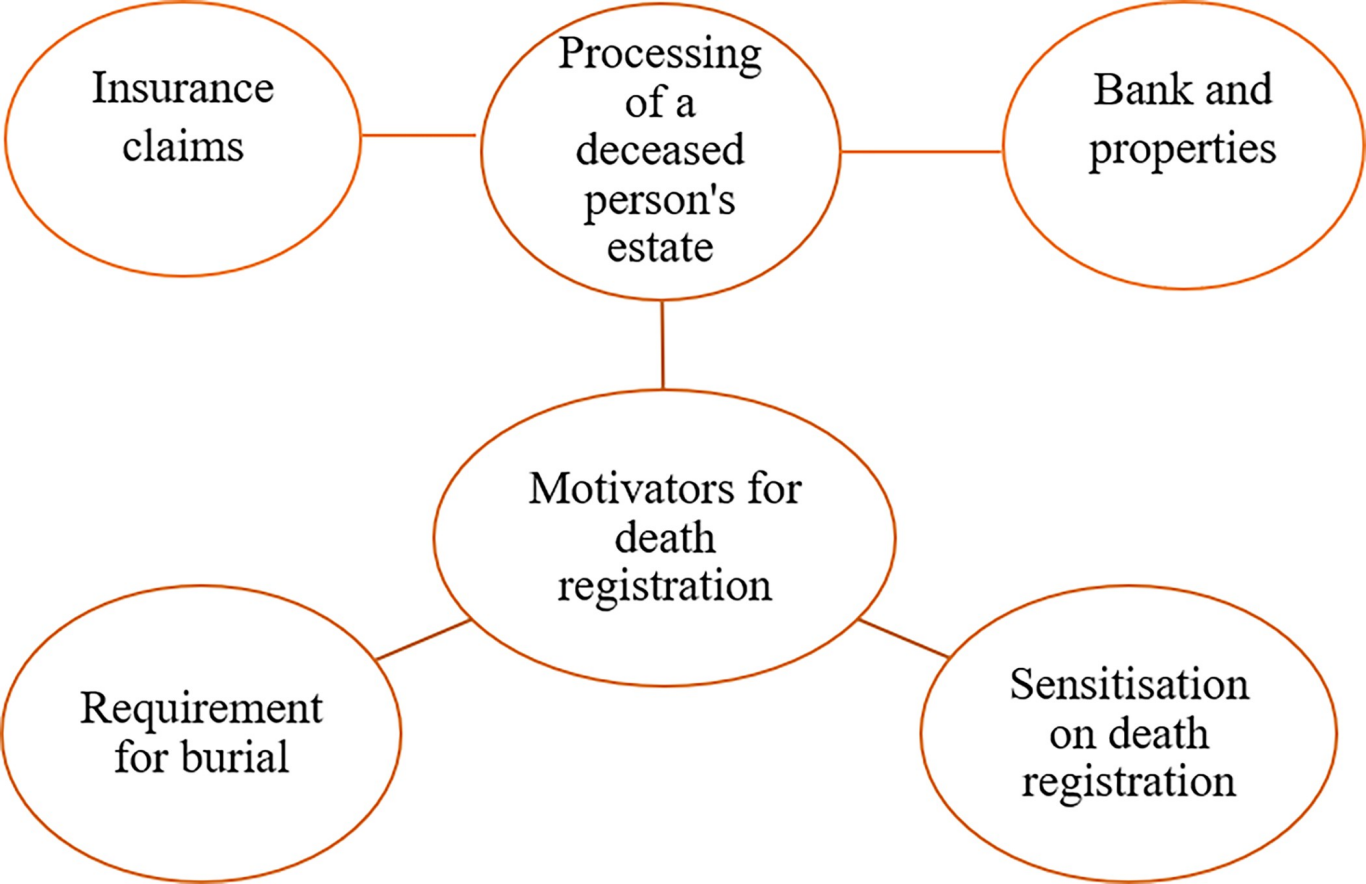
Thematic network showing motivators for death registration.

Three main themes were identified regarding the reasons for death certificate registration. They were (a) processing of a deceased person’s estate, (b) sensitisation on death registration, and (c) requirements for the burial of a dead person. Fig 3 shows the thematic diagram of motivators for death registration.

*Processing of a deceased person’s estate*. The processing of a deceased person’s estate inspired people to register the death of their relatives. These estates include assets such as bank accounts as well as insurance benefits. Regarding assets, interviewees emphasised that the need to make claims for a dead person’s assets motivates people to register their dead relatives for a death certificate. They expressed that they use death certificates to claim money at the bank and properties, as well as claims of insurance benefits.

Some interviewees explained that a death certificate is an important document needed to claim money from the bank account(s) of a dead person. It was revealed that the death certificate is used to acquire a letter of administration from the court before being taken to the bank to claim a deceased person’s money. Without the death certificate and the letter of administration, no money can be withdrawn from the dead person’s account.

"*They mostly register due to claims. The deceased person has some money at the bank, so they will need the dead certificate and also do letters of administration to claim the money from the bank."* (R1, Female)

There were instances where some interviewees mentioned that deaths are registered to facilitate the process of the acquisition of wills or properties of the deceased. Some interviewees expressed that a death certificate is the main document needed to inherit a dead person’s property. As a respondent reiterated, when no property is involved, most people do not want to register for the death certificate. The registration is hastened when the deceased is a prominent person who left enough property. Therefore, there is a time limit for the registration to claim the properties of a dead person.

"*I think when there is a property to share, they come and do the death certificate. Apart from that, I do not know why they do not do the death certificate here."* (R8, Female)

"*It is these two things*, *claims and wills issues*. *The primary document required for the whole process is a death certificate*. *So anytime a death occurs*, *especially when the person is prominent*, *the family knows they have to get these things in order because there is also a time when you cannot also register the death anymore*.*"* (R5, Male)

Furthermore, some interviewees expressed that claiming insurance benefits for a deceased is another reason for the acquisition of death certificates. Mostly, some individuals take out funeral insurance policies on behalf of parents or loved ones, so when the person dies, they need a death certificate to process the insurance claims. Due to its attendant financial burden, these individuals take out these funeral policies to enable them to cater to funeral emergencies.

"*Most of the time, some people do funeral policies for their parents. That also helps those who have registered for them to get that hamper of money when something happens. That also helps. Again, it is not only those who are dead at the hospital that they give the documents for you to go and bury them. When you are involved in an accident, or you sleep and do not wake up. There are ways that you can do all these things for us to see and register for the burial permit so that it will be buried.*" (R6, Male)

"*So now*, *it’s gradually improved*. *Due to these insurance companies and other factors*, *people insure their fathers and mothers*. *When the person dies*, *you have to come*. *You know*, *without the death certificate*, *they won’t get anything*, *and those whose parents were civil servants*, *when their parents die*, *they won’t get anything from either SSNIT or the bank*, *so I think it has gradually improved*.*"* (R10, Male)

In addition, an interviewee indicated that although most people do not know the relevance of the death certificate, they register for it when they want to make claims for a deceased person on their insurance policy.

"*Some do not know its importance unless they want to claim something. Perhaps the deceased person is on one of the policies they want to claim. They will rush for that one. You will not even follow up on it, but they will rush for it.*" (R12, Female)

*Sensitisation on death registration*. Some interviewees recounted that public sensitisation has helped to increase death registration. While processing a deceased person’s estate may be the most pressing factor for death registration, some of the increase in registration rate could be attributed to general sensitisation efforts on the uses and benefits of death registration.

"*Some people are sensitised to know that they have to register the deceased before you bury them while others are already aware that they have to register their dead relatives whether they have to make claims or not."* (R2, Male)

"***I***: *Some of the reasons for death registration in this municipality*? ***R***: *Death registration is for statistical purposes because some people are aware*, *they tend to register the death*.*"* (R5, Male)

*Requirement for burial*. Another factor that motivates people to register a deceased for a death certificate is the requirement for burial of the dead body. In Ghana, most cemeteries require a burial permit or a death certificate; hence, without either of these, you will be disallowed to bury a dead person. In such situations, people go for the burial permit first to bury a dead person, which is then followed by a dead certificate.

"*Some of the cemeteries, without burial permits, they won’t grant you permission to bury your dead relative. This forces or pushes them to come and do the registration before they bury the deceased"* (R2, Male)

"***I*:** *Therefore*, *what are some of the factors that motivate people to register for death certificates*? *R*: *You cannot go to Awudome cemetery without the death extract*.*"* (R4, Male)

"*Some people also do the registration for a burial permit and later come back to do a death certificate because there is a difference when you register for a burial permit*. *The burial permit is the only certificate you require to bury*. *When you do not have it*, *you cannot bury it*.*"* (R6, Male)

#### Barriers to death registration

**Fig 4:**
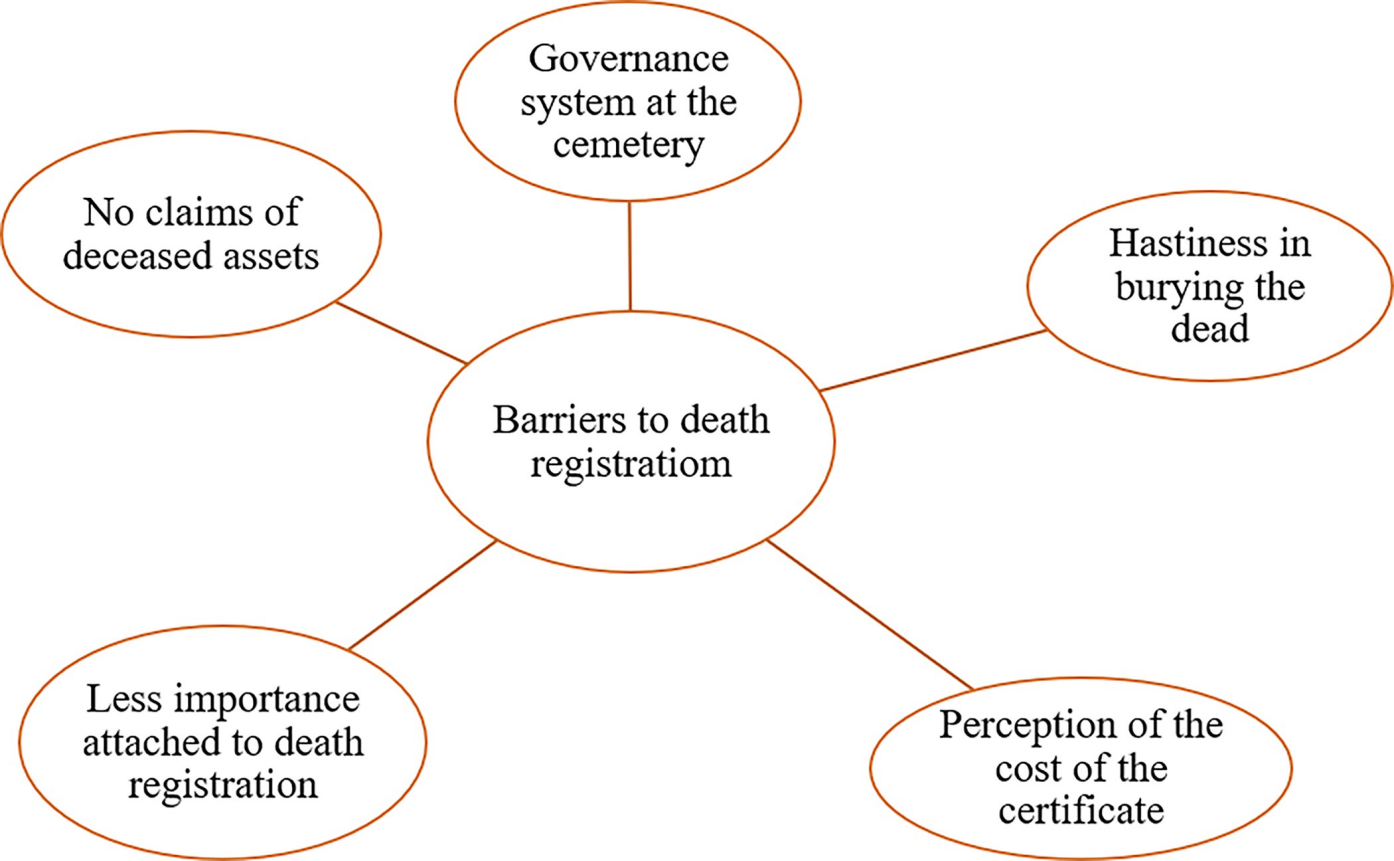
Thematic network showing barriers to death registration.

Five (5) main basic themes were identified under the reasons for not registering a deceased for a death certificate. These are the governance system at the cemetery, the hastiness in burying the dead, the perception of the cost of the death certificate, less importance attached to death registration, and no claims of deceased assets. Fig 4 shows the thematic network of the barriers to death registration.

*Governance system at the cemetery*. Bribing sexton has become a phenomenon attributed to the reasons for non-registration for a death certificate or burial permit. Relatives of the deceased without either a burial permit or death certificate bribe the sexton for them to be given a place to bury the dead. Bribery occurs due to the absence of checks and balances to guarantee proper conduct.

"*Somebody might have died at home; then, they will prepare him and go and bury him. You will be there, and then they will inform you that this person is dead, but they have left to bury him. When they got there, the watchman asked for their permit. They tell him they do not have a permit. Therefore, he should take 100 Ghana Cedis (US $10) for them to bury the deceased person.* (R6, Male)

In addition, the governance of our cemetery system also contributes to the non-registration of a deceased for a death certificate. Most of the cemeteries are owned by the indigenes, so they sometimes determine what is done, which limits the power of birth and death registration officers to insist on the registration of the dead for a death certificate. This situation is very common in rural areas or villages where customs and traditions are strictly adhered to, and there are no claims for insurance or property. However, an interviewee indicated that they enforced the law to ensure that deaths from other villages and towns required a burial permit and that they were registered for a death certificate.

"*In this community, the indigenes own the cemeteries. The lands are for them, so they choose to bury without any permits from anywhere. It is so common in the typical villages."* (R2, Male)

"*They will bury at the village; they would not get the information*, *and there are no checks or balances*. *They are small communities where the dead can easily be buried without our knowledge*. ***I*:** *Therefore*, *your only issue is when the body was not deposited in this morgue*. ***R*:** *Yes*, *it will not be buried here at the Sunyani Municipal*, *Sunyani cemetery*. *Before you take it [body] out*, *you will get the burial permit so we can capture them*.*"* (R6, Male)

*Hastiness in burying the dead*. One interviewee mentioned that sometimes people are in a hurry to bury the dead, so they do not register. This situation is mostly associated with Muslims, who are required by religion to bury a dead person within 24 hours of death. In some cemeteries where the requirements are strictly enforced, the relatives who have failed to secure a burial permit but are in haste to bury their relatives are forced to leave behind the corpse and acquire the burial permit before they can return to proceed with the burial.

"*I sometimes think when somebody or their family member is deceased, they are so hot-headed, they are ’wild,’ so they don’t have time. They do not include the registration in their plans until the day of burial when they get to the cemetery, and those who take care of the cemetery ask for a burial permit, which is when they return. They leave the body there, rush back. For the Muslims, we understand them because when the person dies today, they have to bury them tomorrow, so we are always available to do that for them."* (R2, Male)

*Perception of the cost of a death certificate*. Interviewees described the perception of the cost of a certificate as one of the factors that demotivate people from registering a dead person. There is a perception that a death certificate is very expensive and that it is not easy to obtain a certificate. This situation stops people from initiating the death registration process. However, there is no cost for early registration of death certificates.

"*Some have the notion that it’s an expensive thing to do while it’s not so. Sometimes when they come and you tell them it is free so they should come and register, and they register."* (R2, Male)

*Less importance attached to death certificate*. Less importance associated with the death certificate was identified as one of the barriers to death registration. The interviewees expressed that most people do not know the relevance of death certificates. Hence, they do not see the need to register for a death certificate. Despite interventions such as sensitisation on the need to register a dead person, people still refuse to register the death of their relatives or loved ones.

"*Here, they do not value death certificates. They do not see its importance. … Some will tell you the person is dead, so why should I register the death? You see, unless you take your time as an officer to explain things to the person. Consequently, we have to let them know that it is very important that when someone dies, the deceased is registered."* (R9, Male)

"*In this district*, *death registration is another problem*. *Many people do not value the importance of a death certificate*. *When someone dies*, *they do not see the need to register the death*.*"* (R11, Male).

One interviewee noted that sometimes it is very difficult to educate people on the relevance of death certificates, unlike birth certificates, which also makes it very difficult for people to understand the relevance of death registration of their dead relative.

"*They do not see the point of death registration, yes, and it is also difficult to sensitise these people because, with the births, they will come for weighing [child welfare clinics]. There is a gathering of the new mothers. But you hardly get the gathering of people who have lost their family members or friends, so that makes it hard to gather a group of people to educate them on why they should register their deaths. Therefore, I will say that that is a challenge to us"* (R5, Male)

*No claims of deceased assets*. According to interviewees ’ narratives, most people do not register for a death certificate when there is no claim of deceased assets. With this, they know the registration process but do not see the need to register for the certificate when there are no assets at stake to claim.

"*When they are done, and there is no claim, they do not come for a death certificate. The certified copy, they don’t."* (R1, Female)

"***I*:** *What prevents people from registering for death certificates*? ***R*:** *Maybe at times*, *if the person dies at home and probably has nothing to his name*, *the dead person has nothing*. *If he has no property*, *maybe his children or family can claim it*.*"* (R3, Male)

"*Most of them*, *it is like a local family; there is nothing to share*. *We know what belongs to us*, *so why register for a death certificate*? *It is not important*. *Unless a situation requires a death certificate before they begin to think about it*.*"* (R7, Female)

## Discussion

This study applied a qualitative method to examine the motivations for and barriers to death registration in Ghana. Most of the interviewees interviewed had a tertiary education and had worked for at least 10 years. Narratives from the birth and death registration officers revealed a general trend of death registration in Ghana. Most of the interviewees highlighted that death registration was low. Although they acknowledged that there has been an improvement in the registration of deaths, they explained that it continues to be generally low in almost all the districts. The low death registration was attributed to the long distance to district offices, the inadequate registration offices, and the nature of our cemetery system. The findings of this study are consistent with other studies that cited accessibility as one of the main reasons for the low registration of deaths [3,8,19]. In this study, accessibility to registration offices is a key factor since there are few registration offices in each district. In each district, there is one main office and several satellite offices. People must travel long distances, especially those in villages and isolated communities, to district offices to register a death. Long distance and transportation costs sometimes demotivate people to travel to the district offices to register their dead relatives. It is also worth noting that poverty is disproportionately high in rural areas, including villages and isolated communities [28–30], which exacerbates their plights.

The study indicated that factors such as processing of a deceased person’s estate, obligation to register, and requirement for burial motivate people to register for death. Among these factors, most interviewees mentioned the processing of a deceased person’s estate as the main reason for death registration. Regarding the processing of a deceased person’s estate, most of the interviewees emphasised that the need to claim the property of a dead person motivates relatives to register for a burial and a death certificate. The findings of this study are consistent with studies in Senegal [3] and Uganda [9], which reported that inheritance is a key motivator for death registration. The probable reason could be that the family of a dead person needs a death certificate as a key document to process claims such as insurance and deposits of the dead person. In Ghana, some people have insurance for themselves, their parents, or their relatives [31]. Therefore, the death of a person necessitates the need for a death certificate to process the claims of the deceased.

Though a death certificate is a requirement for burial in Ghana, the accounts of interviewees revealed that relatives of deceased persons are able to bribe sexton to ensure they bury their deceased relatives without one. These findings highlight the need to enhance monitoring mechanisms at cemeteries to supervise the actions of cemetery staff and provide frequent training on ethical behaviour, including the acceptance of bribes.

The obligation to register for death was identified as a motivator of death registration. The study showed that some people are sensitised and aware of the need to register deaths. They tend to register for the death of their loved ones whether there is compensation or not. Such registration tends to help improve the data quality of death registration in Ghana. This finding is consistent with a study by Atuhaire et al. [9] in Uganda, which reported that people register for a death certificate because it is a legal requirement. Therefore, they are obliged to do so. Policymakers should strengthen their public awareness campaigns about the need for death registration to increase death registration nationwide.

In addition, the findings of the study showed that the governance system at the cemetery, the hastiness in burying the dead, the perception of the cost of the death certificate, less importance attached to death registration, and no claims of deceased assets were barriers to dead registration in Ghana.

The governance system at cemeteries in rural areas promotes the non-registration of deaths. Compared to cemeteries in urban areas, people who reside in rural areas are often the owners of the land and adhere to their customs and beliefs, which makes it a challenge for government and non-governmental agencies to put measures to regulate the cemetery to ensure that all deaths are duly registered. The results of this study are similar to those of other studies [19] that found that cultural and governance systems limit people’s ability to register for death. This finding calls for collaboration between policymakers and traditional authorities, including chiefs, queens, and clan heads, to educate community members about the need to register deaths and enforce laws on death registration.

Consistent with other studies [20,32], less importance attached to death certificates was identified as a hindrance to death registration. Some people were ignorant about the relevance of death certificates [2]. Also, some interviewees mentioned that it was difficult to educate people about death registration compared to birth registration. This finding highlights the need to provide regular training workshops for birth and death registration officers to equip them with the requisite skills and effective strategies to educate the general public about the importance of death registration.

Similar to other studies, the cost associated with death registration was identified as a factor that demotivates other people to register a death [32]. In this study, interviewees indicated that the perceived cost of death registration limited its patronage. Some persons perceived death registration as expensive, which demotivated them to register their dead relatives and loved ones. The Births and Deaths Registry should consistently advertise the pricing of its services, which would aid in rectifying any misconceptions regarding the cost of its services.

In addition, this study revealed that the absence of claims to assets belonging to deceased individuals acts as a demotivator for people to register deaths. This finding emphasises the impact of extrinsic motivation, specifically rewards, on the death registration process. Furthermore, interviewees reported that the hastiness in burying the dead discouraged the death registration. They cited that Muslims have a religious obligation to bury their departed loved ones within 24 hours, which leads them to be prompt in burying their deceased loved ones who are Muslims. This finding supports a previous study [33], which highlights the influence of religious practices on death registration. Habaasa’s [33] study in Uganda found that the religious practice of same-day burial discourages death registration. Although provisions exist for registering deaths after burial, people often lack the motivation to do so, as they may not see its relevance once the deceased has been buried.

## Strengths and limitations of the study

The main strength of this study is its coverage of districts in the three ecological zones in Ghana, which highlights the situation of death registration across the country. However, this study captured only the perspectives of birth and death registration officers, which may not capture all the possible factors that motivate and serve as barriers to the registration of deaths in Ghana. Future studies should interview other stakeholders involved in birth and death registration, such as parents or guardians and opinion leaders, to enhance understanding of the nuances of death registration in Ghana.

## Conclusions

In summary, this study highlighted the barriers to and motivations for death registration in Ghana. The factors that motivate people to register the dead include claims of deceased assets, obligation to register, and requirement for burial. In contrast, factors such as the governance system at the cemetery, the hastiness in burying the dead, the perception of the cost of the death certificate, and no claims of deceased assets demotivate people to register their deceased. Policymakers should consider these factors in the design of interventions to increase the uptake of death registration in Ghana.

## Supporting information

S1 Appendix Themes for motivations and barriers to death registration in Ghana. (DOCX)
