# Supplementary material for: "Do we register our deaths?": Motivations and barriers to death registration in Ghana
Source: PLoS One. 2024 Oct 24;19(10):e0312353. doi: 10.1371/journal.pone.0312353 (PMC11500923; doi:10.1371/journal.pone.0312353)
Supplement: S1 Appendix — (DOCX) [file pone.0312353.s001.docx]

**Themes for motivations and barriers to death registration in Ghana**

| **Global themes** | **Organising themes** | **Basic themes** | **Sub-basic themes** | **Sample quotes** |
| --- | --- | --- | --- | --- |
| Factors associated with death registration | Trends in death registration in Ghana | Increased death registration |  | "**I:** How is death registration in this district? **R:** Yes. It is fine. **I:** Has it increased or decreased? **R**: It has increased. In fact, the system or the cemetery is helping. They have an organised system here at the cemetery and the morgues. They have encouraged people to register their deaths before burial." (R5, Male)  **"**Death registration is high because in this place [district], the number of people who are buried outside this district is 1 percent. Most of the people who die here are buried either in Korle Gorno or Awudome, so you cannot escape. You will have to register definitely. Additionally, because of [public] education, we give to the communities, and all that accounts for the increase." (R3, Male)  "Okay, I would say yes, it is better than previous times because right now we are in a kind of a state where before we process any kind of document for the death of an individual, the primary document you need is the death certificate so that encourages people to chase the death certificate **I:** okay **R:** so, it has greatly improved **I:** so, looking at the trend it has increased R: it has increased." (R6, Male) |
|  |  | Low level of death registration |  | "Here, death registration is low, I mean, I do not know how to say it, but it is not plenty. In a month, you can get like 5 or something like that." (R7, Female)  "**I**: Is death registration high or low in the district? **R:** I will say it's low. **I**: Why do you think it's low? **R**: The factors I gave, you know, somebody travelling from a village in Agortor to do registration and, say, our district office registry at Asutsuare. The transportation cost for the return trip will cost the person 100 Ghana Cedis. They get discouraged to travel to (laughs) do those things." (R1, Female)  "**R**: It's very poor because they do not value death certificates here. They do not know its importance. Even though sensitization is ongoing, some residents will tell you the person is dead, so what am I going to register the death for? You see, unless you take your time as an officer to explain things to the person, they would not appreciate its importance." (R9, Male) |
|  | Motivators for death registration | Requirement for burial |  | "Some of the cemeteries, without burial permits, they won't grant you permission to bury your dead relative. This forces or pushes them to come and do the registration before they bury, mmm" (R2, Male)  "**I:** Therefore, what are some of the factors that motivate people to register for death certificates? R: You cannot go to Awudome cemetery without the death extract." (R4, Male)  "Some people also do the registration for a burial permit and later come back to do a death certificate because there is a difference when you register for a burial permit. The burial permit is the only certificate you require to bury. When you do not have it, you cannot bury it.” (R6, Male) |
|  |  | Sensitisation on death registration |  | "Those are the factors. Some are sensitised to know that they have to register the deceased before you bury them while others are already aware that they have to register their dead relatives whether they have to make claims or not." (R2, Male)  "**I**: Some of the reasons for death registration in this municipality? **R**: Death registration is for statistical purposes because some people are aware, they tend to register the death." (R5, Male) |
|  |  | Processing of a deceased person's estate | Bank and properties | "They mostly register due to claims. The deceased person has some money at the bank, so they will need the dead certificate and maybe do letters of administration to claim the money from the bank." (R1, Female)  "I think when there is a property to share, they come and do the death certificate. Apart from that, I do not know why they do not do the death certificate here." (R8, Female)  "It is these two things, claims and wills issues. The primary document required for the whole process is a death certificate. So anytime a death occurs, especially when the person is prominent, the family knows they have to get these things in order because there is also a time when you cannot also register the death anymore." (R5, Male) |
|  |  |  | Insurance benefits | **"**Most of the time, some people do funeral policies for their parents. That also helps those who have registered for them to get that hamper of money when something happens. That also helps. Again, it is not only those who are dead at the hospital that they give the documents for you to go and bury them. When you are involved in an accident, or you sleep and do not wake up. There are some ways that you can do all these things for us to see and register for the burial permit so that it will be buried." (R6, Male)  "So now, it's gradually improved. Due to these insurance companies and other factors, people insure their fathers and mothers. When the person dies, you have to come. You know, without the death certificate, they won't get anything, and those whose parents were civil servants, when their parents die, they won't get anything from either SSNIT or the bank, so I think it has gradually improved." (R10, Male)  "Some do not know its importance unless they want to claim something. Perhaps the deceased person is on one of the policies they want to claim. They will rush for that one. You will not even follow up on it, but they will rush for it." (R12, Female) |
|  | Barriers to death registration | Governance of the cemetery |  | "Somebody might have died at home; then, they will prepare him and go and bury him. You will be there, and then they will inform you that this person is dead, but they have left to bury him. When they got there, the watchman asked for their permit. They tell him they do not have a permit. Therefore, he should take 100 Ghana Cedis ($10) from them to allow them to bury the deceased person (R6, Male)  "In this community, the indigenes own the cemeteries. The lands are for them, so they choose to bury without any permits from anywhere. It is so common in the typical villages." (R2, Male)  "They will bury at the village; they would not get the information, and there are no checks or balances. They are small communities where the dead can easily be buried without our knowledge. **I:** Therefore, your only issue is when the body was not deposited in this morgue. **R:** Yes, it will not be buried here at the Sunyani Municipal, Sunyani cemetery. Before you take it [body] out, you will get the burial permit so we can capture them." (R6, Male) |
|  |  | Hastiness to bury the dead |  | "I sometimes think when somebody or their family member is deceased, they are so hot-headed, they are 'wild,' so they don't have time. Hahaha. They don't include the registration in their plans until the day of burial when they get to the cemetery, and those who take care of the cemetery ask for a burial permit, which is when they return. They leave the body there, rush back, and then especially the Muslims for them, we understand them because when the person dies today, they have to bury them tomorrow, so always we are available to do that for them." (R2, Male) |
|  |  | Perception of the cost of the certificate |  | "Some have the notion that it's an expensive thing to do while it's not so. Sometimes when they come and you tell them it is free so they should come and register, and they register." (R2, Male) |
|  |  | Less importance is attached to death registration |  | "Here, they do not value death certificates. They do not see its importance. … Some will tell you the person is dead, so why should I register the death? You see unless you take your time as an officer to explain things to the person. If I don't have the use of it, so the person is dead, I should go and put it down. So that one affects them, so we have to let them know that it is very important that when someone dies, you have to register." (R9, Male)  "In this district, death registration is another problem. Many people do not value the importance of a death certificate. When someone dies, they do not see the need to register the death” (R11, Male)  "They do not see the point of registering the death of someone, yes, and it is also difficult to sensitise these people because, with the births, they will come for weighing [child welfare clinics]. There is a gathering of the new mothers, but you hardly get the gathering of people who have lost their family members or friends, so that makes it hard to gather a group of people to educate them on why they should register their deaths. Therefore, I will say that that is a challenge to us" (R5, Male) |
|  |  | No claims of deceased assets |  | "When they are done, and there is no claim, they do not come for a death certificate. The certified copy, they don't." (R1, Female)  "**I:** What prevents people from registering for death certificates? **R:** Maybe at times, if the person dies at home and probably has nothing to his name, the dead person has nothing. If he has no property, maybe his children or family can claim it”. (R3, Male)  "Most of them, it is like a local family; there is nothing to share. We know what belongs to us, so why register for a death certificate? It is not important. Unless a situation requires a death certificate before they begin to think about it." (R7, Female) |
